# Mechanisms of U87 Astrocytoma Cell Uptake and Trafficking of Monomeric versus Protofibril Alzheimer’s Disease Amyloid-β Proteins

**DOI:** 10.1371/journal.pone.0099939

**Published:** 2014-06-18

**Authors:** Yali Li, Deshu Cheng, Ran Cheng, Xinyu Zhu, Tao Wan, Jianmiao Liu, Rongying Zhang

**Affiliations:** Key Laboratory of Molecular Biophysics of the Ministry of Education, School of Life Science and Technology, Huazhong University of Science and Technology, Wuhan, Hubei, China; Federal University of Rio de Janeiro, Brazil

## Abstract

A significant hallmark of Alzheimer’s disease is the formation of senile plaques in the brain due to the unbalanced levels of amyloid-beta (Aβ). However, although how Aβ is produced from amyloid precursor proteins is well understood, little is known regarding the clearance and metabolism of various Aβ aggregates from the brain. Similarly, little is known regarding how astrocytes internalize and degrade Aβ, although astrocytes are known to play an important role in plaque maintenance and Aβ clearance. The objective of this study is to investigate the cellular mechanisms that mediate the internalization of soluble monomeric versus oligomeric Aβ by astrocytes. We used a combination of laser confocal microscopy and genetic and pharmacological experiments to dissect the internalization of sAβ42 and oAβ42 and their postendocytic transport by U87 human brain astrocytoma cell line. Both Aβ42 species were internalized by U87 cells through fluid phase macropinocytosis, which required dynamin 2. Depleting LDL receptor-related protein 1 (LRP1) decreased sAβ42 uptake more significantly than that of oAβ42. We finally show that both Aβ42 species were rapidly transported to lysosomes through an endolytic pathway and subjected to proteolysis after internalization, which had no significant toxic effects to the U87 cells under relatively low concentrations. We propose that macropinocytic sAβ42 and oAβ42 uptake and their subsequent proteolytic degradation in astroglial cells is a significant mechanism underlying Aβ clearance from the extracellular milieu. Understanding the molecular events involved in astrocytic Aβ internalization may identify potential therapeutic targets for Alzheimer’s disease.

## Introduction

Senile plaques in the brain are one of the hallmarks of Alzheimer’s disease (AD). The main component of these senile plaques is amyloid-beta (Aβ), a metabolic product of amyloid precursor protein (APP). Steady-state levels of Aβ in the normal brain are maintained by a balance between its production and clearance. However, this balance is broken in the AD brain due to either Aβ overproduction or reduced Aβ clearance. Thus, Aβ can accumulate in the brain and form amyloid plaques that cause dementia and neurodegeneration [1]. It has been reported that only 5% of AD cases (familial type) is due to Aβ overproduction arising from mutations in the APP gene or in APP processing enzymes, whereas the majority (95%) of so-called sporadic AD cases are likely caused by dysfunctions in Aβ solubility, endocytosis, degradation, transcytosis, and removal [2]. However, despite the dramatic progress that has been achieved in understanding how Aβ is produced from APP, the mechanisms of Aβ aggregation, clearance from the brain, and metabolism remain unclear [3].

The AD brain contains soluble and insoluble assemblies of Aβ, both of which have been hypothesized to underlie dementia [4]. Insoluble fibrillar forms of Aβ arise from the polymerization of the soluble, monomeric, or oligomeric forms of Aβ peptides. Early evidence for Aβ-induced neurotoxicity in cell culture and *in vivo* was associated with fibrillar forms, such as those observed in neuritic (amyloid) plaques. Recent studies have highlighted that natural as well as synthesized Aβ42 oligomers (oAβ42) and immature fibrils exert much greater toxic effects on neurons and disrupt learned behavior in a rapid, potent, and transient manner [5], [6], [7]. A central message from these studies is that subtle brain dysfunction occurs in the presymptomatic stages of AD that may be related to Aβ oligomer effects; therefore, these effects may be reversible with appropriate interventions before widespread neuronal degeneration occurs.

One of the main questions under debate concerning Aβ toxicity is the (sub-) cellular localization of action. In addition to the deposition of Aβ peptides into extracellular plaques, numerous studies have provided evidence for the presence of Aβ within neurons in post-mortem AD and transgenic mouse brains [8]. It is unknown whether intraneuronal Aβ originates from the retention and subsequent aggregation of intracellularly generated Aβ or from the reuptake of extracellular Aβ. The accumulation of activated microglial cells and astrocytes close to Aβ deposits suggests that these cells play a role in AD pathology.

Microglial cells are mononuclear phagocytes of the innate immune system in the central nervous system (CNS). These cells reportedly mediate the clearance of fibrillar Aβ (fAβ) through receptor-mediated phagocytosis and internalize soluble Aβ (sAβ) from the extracellular milieu through a nonsaturable, fluid phase macropinocytic mechanism [9]. Internalized Aβ subsequently undergoes proteolytic degradation in late endolysosomal compartments, which suggests a neuroprotective role for microglial cells via their ability to internalize and degrade Aβ [10], [11], [12], [13].

However, astrocytes are the most abundant cell type in the CNS. Nielsen *et al.* [14], [15] showed that primary human astrocytes in culture could bind to and ingest Aβ, which supported the assumption that astrocytes played an important role in plaque maintenance and Aβ clearance. Mulder *et al.* [16] examined the expression of the potential rodent astrocytic Aβ-receptors SCARB1, MARCO, and LRP2 by cultured primary human astrocytes isolated from brain specimens of non-demented control subjects and AD patients. However, little is known about the astrocyte endocytic mechanism and which receptor(s) mediate the uptake of Aβ, particularly for oligomeric Aβ in astrocytic cells [4], [17].

Taken together, the regulation of sAβ levels is a critical determinant in the development of AD pathology. In this study, we focused on how astrocytes, the major glial cell type in the CNS, participated in maintaining Aβ homeostasis. Because the aggregation status (solubility) of Aβ goes hand in hand with its clearance [15], we systematically compared the uptake and postendocytic trafficking of soluble monomeric amyloid-beta protein (sAβ) and protofibril (PF), an oligomeric assembly of Aβ (referred to here as oAβ). We studied Aβ42, because several lines of evidence show that it is the Aβ42 peptide other than Aβ40, which is observed in neurons [4], [18], [19]. We demonstrate macropinocytic sAβ42 and oAβ42 uptake and their subsequent proteolytic degradation in U87 cells, which may represent a significant mechanism that underlies Aβ clearance from the extracellular milieu. Although several reports have suggested that lipoprotein-related protein 1 (LRP1)-mediated Aβ uptake in glial cells, to the best of our knowledge, our study is the first to provide direct evidence that LRP1 is differentially involved in the internalization of the monomeric and oligomeric forms of Aβ42 by U87 cells. Furthermore, we quantified the degradation for these two forms of Aβ42 peptides in U87 cells, which may constitute a risk factor for senile plaque formation. This is relevant to developing an appropriate therapeutic approach for AD.

## Materials and Methods

### Reagents and Plasmids

HiLyte Fluor555-labeled Aβ42 was from Anaspec (Fremont, CA, USA). High glucose Dulbecco’s modified Eagle Medium (DMEM), fetal bovine serum (FBS), Opti-MEM (OMEM), Lipofectamine 2000, Alexa568-Tfn and LysoTracker Green were from Invitrogen (Shanghai, China). Amiloride hydrochloride hydrate, Methyl-β-cyclodextrin, wortmannin, Saponin, Thioflavin T and DAPI were from Sigma-Aldrich (St. Louis, MO, USA). CellTilter 96® AQ_ueous_ One Solution Cell Proliferation Assay (MTS) kit was purchased from Promega (Madison, USA). Dynasore was a kind gift from Dr. Thomas Kirchhausen (Harvard Medical School, Boston, MA). Polyclonal rabbit anti-LRP1 was from Abclonal (Wuhan, China); Monoclonal mouse anti-α-tubulin was from Abcam (Cambridge, UK). HRP-conjugated Affinipure goat anti-mouse and goat anti-rabbit secondary antibodies were from ProteinTech Group (Chicago, USA). SuperSignal West Femto Maximum Sensitivity Substrate was from Thermo scientific (Waltham, MA, USA).

The 21-nucleotide target sequence of human LRP1 gene (^88^AAGCAGTTTGCCTGCAGAGAT^108^) was used to generate the plasmid encoding shRNA against LRP1 [20]. Dynamin (dyn) mutants, including dyn1K44A and dyn2K44A, were generated by exchanging the nucleotide sequence encoding lysine 44 with a homologous fragment encoding alanine 44. The EGFP-Rab5 plasmid was a kind gift from Dr. Emmanuel Boucrot (MRC Laboratory of Molecular Biology, Cambridge, UK).

### Preparation of Aβ42 Monomers and Oligomers

Lyophilized HiLyte Fluor555-labeled Aβ42 was dissolved in 0.1% NH_4_OH to get soluble monomeric Aβ42 (sAβ42) at a concentration of 400 µM. Aliquots were either stored at 4°C as sAβ42 stocking solution or used for preparing oAβ42. The procedure was to dilute the stock sAβ42 solution to a concentration of 100 µM in 150 mM NaCl buffered at 10 mM Tris (pH 7.4), subsequently 0.1 M HCl was added to adjust the pH to 2.0, and the sample solution was incubated at 37°C for 24 h with mild agitation. The pellets were collected by centrifuging the sample at 12,000 rpm for 20 min and resuspended in PBS to a final concentration of 100 µM and stored at 4°C. When used, the working concentration of the peptides was adjusted to 0.4 µM. Unlabeled synthetic human amyloid-β peptide was identically oligomerized with the above protocol and used for experiments.

### Characterization of Oligomer Preparations

#### Electron microscopy

Aβ42 oligomers were applied to formvar-coated 300-mesh copper grids for 2 min and excess fluid was filtered off. The samples were then stained with 1% uranyl acetate for 1 min, excess fluid was filtered off and the grids were examined with H-8100 transmission electron microscope (Hitachi, Tokyo, Japan) operated at 150 KV and 32,000× magnification.

#### ThT fluorescence assay

The assay was performed according to a standard protocol [21]. A 200 µM aqueous Thioflavin T (ThT) was prepared and filtered through 0.22 µm filter. For measurement, Aβ42 samples (monomers or oligomers) were prepared in 10 µM ThT/deionized water solution, pH 7.4. Immediately after mixing, ThT fluorescence of samples was measured with the excitation wavelength at 450 nm and emission wavelength at 482 nm with 470 nm cut-off by a Microplate Reader (Molecular Devices, Sunnyvale, CA, USA).

### Cell Culture and Transfection

Human U87 astrocytoma cell was from Dr. Jianmiao Liu’s lab, and was grown at 37°C with 5% CO_2_ and 100% humidity in DMEM supplemented with 10% FBS, 100 U/ml penicillin and 100 µg/ml streptomycin. Transient transfections of plasmids were performed using Lipofectamine 2000 Reagent Kit (Invitrogen) according to the manufacturer’s instructions, and cells were examined 24 h after. shRNA transfection was also performed using Lipofectamine 2000, cells were subjected to two successive transfection with 48 h interval. Cells were seeded on glass coverslips 12 h after the second transfection and examined 24 h later. Control cells were treated similarly without addition of shRNA.

### Western Blot, Uptake, and Degradation Assays

#### Western blot

U87 cells were transfected with vehicle or LRP1 specific shRNA. 72 h posttransfection, cells were washed twice in PBS buffer and were lysed in lysis buffer (150 mM sodium chloride, 1.0% NP-40, 50 mM Tris, pH 8.0, supplemented with protease inhibitor cocktail). Equal amounts of protein of each sample were subjected to SDS-PAGE followed by membrane transfer. Membrane was incubated with appropriate antibodies, developed by enhanced chemiluminescence regents, and the immunoreactive bands were visualized by autoradiography. For densitometric analyses, immunoreactive bands on films were quantified using ImageJ.

#### Uptake

U87 cells were seeded on coverslips and grown at 37°C overnight in complete medium. Uptake experiments were initiated by incubating cells at 37°C with 0.4 µM HiLyte Fluor555-tagged Aβ42 peptides for 2 h, which were diluted in imaging medium (α-MEM without phenol red supplemented with 20 mM HEPES, pH 7.4, and 5% FBS). Cells were subsequently washed three times with ice-cold PBS, fixed with 3.7% PFA for 20 min and stained with 1 µg/ml DAPI diluted in PBS containing 0.1% saponin for 15 min at room temperature. Then, cells were mounted with a mounting solution containing 50% glycerol and 50% PBS. For endocytosis inhibition experiments, cells were pretreated with various endocytotic inhibitors with indicated times followed by Aβ42 peptides uptake in the presence or absence of these inhibitors.

#### Degradation assay

Cells were incubated in fresh DMEM containing 0.4 µM fluro-tagged Aβ42 monomers or oligomers for 2 h. After extensively wash, cells were chased in fresh completed medium with indicated times up to 72 hours. Cells were subsequently fixed, stained with DAPI and mounted as described above. Images acquisition and analysis were performed as described below.

### Localization of Internalized Aβ42 within the Endolysosomal System

The trafficking of fluro-tagged monomeric and oligomeric Aβ42 was measured as follows. To examine the localization in early endosomes, cells were transfected with EGFP-Rab5 plasmid to label early endosomes. 24 h after transfection, cells were treated with Aβ42 peptides for indicated time periods. After changing with the fresh imaging medium, live cell imaging was then taken using a confocal microscope with an objective heater (set at 37°C). To examine the localization of peptides in lysosomes, plain cells were first treated with Aβ42 peptides for indicated time periods, and 30 min before taking imaging, lysotracker Green were supplemented in the incubation medium (50 nM) to label lysosomes. Afterwards, fresh medium was changed and live cell imaging was taken.

### Confocal Microscopy

All the images were obtained by a spinning disk confocal imaging sysytem (CSU-X1 Nipkow Yokogawa, Japan) under the control of Andor IQ 2.7 software attached to an Olympus IX-71 inverted microscope (Olympus Corp., Japan). An oil-immersion objective (60×NA1.45) was used. Three 50 mW solid-state laser**s** (405 nm, 491 nm, and 561 nm) coupled to an acoustic-optical tunable filter (AOTF) were used as light source to excite DAPI, EGFP, and HiLyte Fluor555. 3D stacks of optical sections spaced 0.2 µm and spanning the complete volume of the cells were acquired. Images were analyzed with ImageJ 1.45 m (Wayne Rasband, National Institutes of Health).

### Data Analysis

#### Uptake assays data

The integrated amount of HiLyte Fluor555-tagged sAβ42 or oAβ42 peptides accumulated within the cell boundaries corrected by background represents the total uptake of ligands, as described before [22]. Briefly, in most cases, the integrated intracellular signals were determined from a 2D projection image of the fluorescence intensities of a Z-stack lacking the most bottom and top planes as they mostly contain plasma membrane signals. The cell outline in each plane was determined by increasing the brightness of the image. In some cases, bright field images were also taken to help identify the cell outlines. The integrated fluorescence within the cell was background-corrected by subtraction of the fluorescence signal surrounding the cell under analysis and its value was then normalized to the mean (average) of the control cells set to 100. For plain cells in the absence or presence of various pharmacological treatment, all intact cells in each image field were used for quantification of Aβ uptake; for cells transfected with plasmids, only the positively transfected cells were used for quantification of Aβ uptake, which was estimated from the EGFP signal in green channel.

#### Degradation assay analysis

Degradation of the internalized sAβ42 or oAβ42 was examined by calculating the normalized signal at each time point as Ii/I0, where I0 is the intensity taken at time point of 0. Estimation of the half-life and rate constants for degradation of Aβ42 peptides were made by fitting data using a single-phase exponential decay equation (Origin, ver. 6.0).

#### Co-localization analysis

Images were deconvolved using AutoQuant, a 50-pixel-wide rolling-ball subtraction algorithm was subsequently used to remove background noise. The sub-stacks of 3 sections spanning 0.4 µm were generated and imported to ImageJ for analysis. Manders’ coefficient was calculated using ImageJ plugin “JACop”. The percentage was scored as the Aβ puncta signals that are positive for EGFP-Rab5 or Lysotracker Green signals with respect to the total number of Aβ puncta.

All data were presented as the mean value ± SEM together with the indicated number of experiments (*n*). Each displayed image was representative of at least three independent experiments.

### Assessment of Cell Viability

Cell viability after treatments with Fluro-tagged sAβ42 or oAβ42 was measured by quantitative colorimetric assay with MTS, as described previously [23]. U87 cells in black 96-well plates at a density of 20,000 cells per well (100 µl) were challenged with sAβ42 or oAβ42 at different concentrations for 24 h or 72 h, and DMSO was used as vehicle control. Cells maintained in the serum free medium without Aβ were used as negative control. Following addition of 20 µl MTS reagent into each well, plates were incubated at 37°C for 4 h. The formazan product was quantified by measuring absorbance at 490 nm with a Microplate Reader (Molecular Devices, Sunnyvale, CA, USA). All the experiments were repeated three times.

## Results

### Preparation and Characterization of sAβ42 and oAβ42

**Figure 1:**
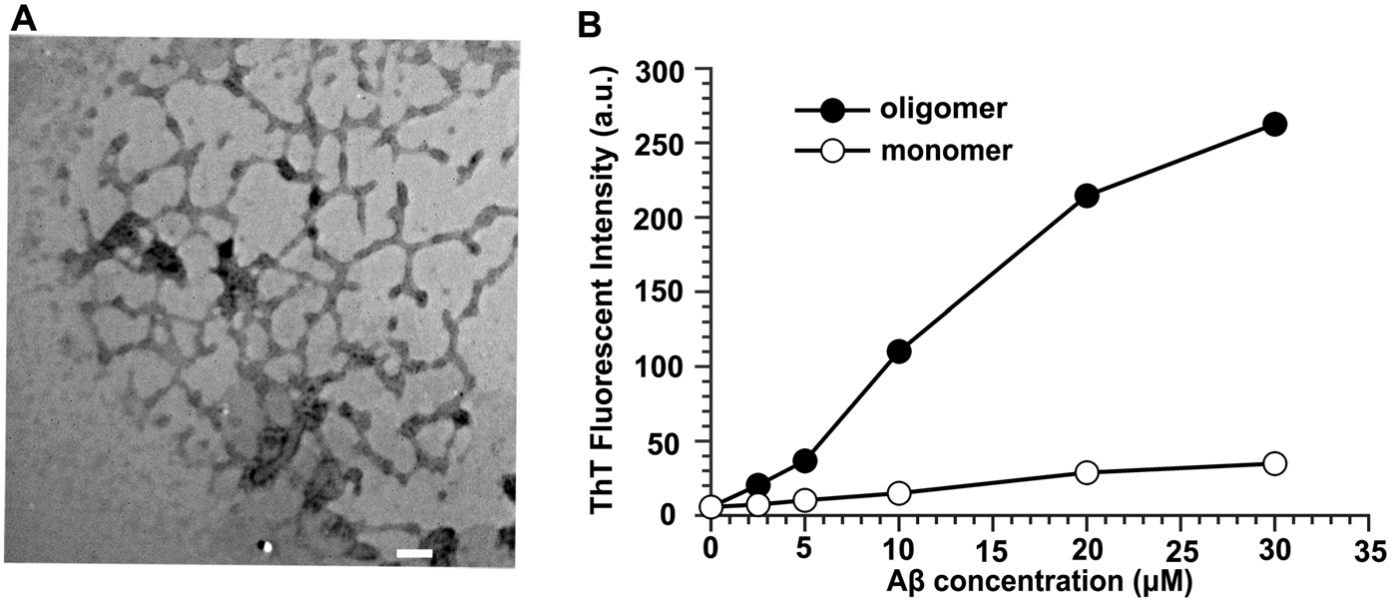
Characterization of oligomeric Aβ42 preparation. (A) Transmission electron micrograph (magnified×32,000) of 100 µM oAβ42. 10 µL of samples were spotted on a glow-discharged, carbon-coated Formavar grid and incubated for 2 min, washed with distilled water, stained with a 1% (w/v) aqueous uranyl acetate solution, and examined using an H-8100 TEM. Scale bar is 100 nm. (B) ThT fluorescence assay of aggregates of Aβ42. a.u., arbitrary unit.

We first prepared homogeneous soluble monomeric Aβ42 (sAβ42), by dissolving the peptide synthetic powder in an NH_3_ solution at pH 12 and with minimal sonication [24]. Furthermore, oligomeric Aβ42 (oAβ42) devoid of mature fibrils was prepared from unlabeled and HiLyte Fluor555-labeled synthetic Aβ42 peptides as described [25]. Transmission electron microscopy (TEM) was used to confirm the oAβ42 preparation. As illustrated in Figure 1A, oligomers appeared as short rod-like structures on electron micrographs with an average length of <150 nm and diameters of approximately 5 nm, in accordance with the previously reported sizes of protofibrils [24], [26].

The fluorescent dye thioflavin T (ThT) is commonly used to detect the formation of amyloid fibrils due to the increases in ThT fluorescence after specifically interacting with amyloid fibrils. However, a recent research demonstrated that oligomeric Aβ contained nanomolar-affinity binding sites for ThT and its analog and suggested that the widely used ThT fluorescence assay for quantifying Aβ fibrils may detect Aβ oligomers [27]. In this study, we used the standard protocol for the ThT assay to further characterize the prepared sAβ42 and oAβ42 (Figure 1B). ThT only exhibited a basal fluorescence level after titration with monomeric Aβ42 solutions of varying concentrations. However, ThT fluorescence increased in a nearly linear-manner after titration with oligomeric Aβ42 solution with concentrations ranging from 0 to 30 µM, which confirmed that these oligomers bound to ThT. These results indicated the successful preparations of sAβ42 and oAβ42, which we used for subsequent experiments.

### oAβ42 Versus sAβ42 Uptake by U87 Cells

**Figure 2:**
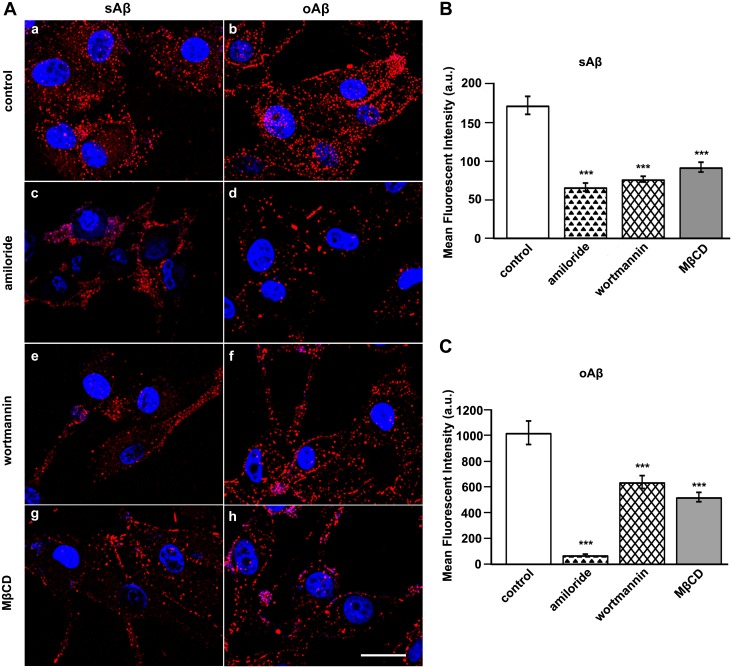
Characterization of sAβ42 and oAβ42 internalization by U87 cells. (A) Representative fluorescent photomicrographs showing the uptake of sAβ42 or oAβ42 by U87 cells that were treated with vehicle, amiloride, wortmannin, or MβCD. U87 cells were pretreated in the absence or presence of 5 mM amiloride for 30 min, 300 nM wortmannin for 1 h, and 1 mM MβCD for 30 min, respectively, followed by incubation with 0.4 µM sAβ42 or oAβ42 for 2 h at 37°C. Subsequently, cells were fixed and analyzed using confocal microscopy for uptake of each Aβ42 as indicated. Nuclei (blue) were stained with DAPI. Scale bar is 20 µm. (B, C) Quantitation of internalized fluorescence-conjugated sAβ42 (B) or oAβ42 (C) in U87 cells based on fluorescence intensity measurements. Values obtained from drug-treated cells were compared to those from mock-treated cells under similar conditions. Results represented the average ± SEM of >25 cells measured in each of three independent experiments. ***p<0.001, t-test.

We used the U87 human brain astrocytoma cell line as a model to study astrocyte uptake of Aβ42 [28]. We first investigated the capability of U87 cells to internalize the monomeric and oligomeric forms of Aβ42. U87 cells were treated with tagged sAβ42 or oAβ42 for the indicated times. After treatment, these cells were washed, fixed, counterstained with DAPI, and observed under a microscope (Figure S1). These results showed that both sAβ42 and oAβ42 were internalized by U87 cells, although the cellular uptake of the latter species appeared to be greater. This was because the average fluorescence intensity of oAβ42 that accumulated within cells was much higher than that of sAβ42, although identical molar concentrations (unit concentration) of these two species were used with U87 cells (results for control in Figure 2; 0.4 µM was used here and for all subsequent internalization experiments). Furthermore, we attempted to identify the mechanistic differences for the astrocyte internalization of sAβ42 and oAβ42.

### sAβ42 and oAβ42 Internalization by U87 Cells Involves Fluid Phase Macropinocytosis and is Sensitive to Reduced Cellular Cholesterol

The cellular mechanisms by which Aβ peptides are internalized with different aggregation states have not been well characterized. To determine the routes of Aβ entry, U87 cells were first treated with amiloride, which is an Na^+^/H^+^-inhibitor commonly used to determine whether the uptake of a particular ligand involves macropinocytosis. Treatment of U87 cells with amiloride significantly reduced their uptake of sAβ42 by 61% and that of oAβ42 by 92%, as determined by internalized fluro-tagged Aβ42s (Figure 2 A, a–d and B). Furthermore, we examined Aβ42 uptake after treating cells with wortmannin, which blocks phosphoinositide 3-kinase (PI3K) activity. PI3K is required for spontaneous cell surface ruffling, which is an integral part of macropinocytosis. As illustrated in Figure 2A (e–f) and the corresponding histograms, sAβ42 and oAβ42 uptake was reduced by 55% and 37%, respectively.

In addition, we determined the effects of removing cholesterol on the internalization of Aβ42 using methyl-β-cyclodextrin (MβCD). Treating U87 cells with MβCD reduced the internalization of sAβ42 by 46% and that of oAβ42 by 48%, which indicated a requirement for cholesterol (Figure 2 A, g and h). A previous report suggested that MβCD promoted Aβ degradation without interfering with its uptake into microglial cells [29]. However, we noticed that the study used flow cytometry to analyze accumulated fluorescently-labeled Aβ, which may not exclude plasma membrane-deposited fluro-tagged ligands, and thus overestimate internalization. Although cholesterol has been considered to be associated with caveolae- or lipid raft-mediated endocytosis, a previous report suggested that as an important component of plasma membranes, cholesterol is necessary for most internalization routes because of its participation in forming an appropriate membrane environment and is necessary for membrane ruffling and actin reorganization [30].

### Dynamin Dependence of Aβ42 Endocytosis by U87 Cells

**Figure 3:**
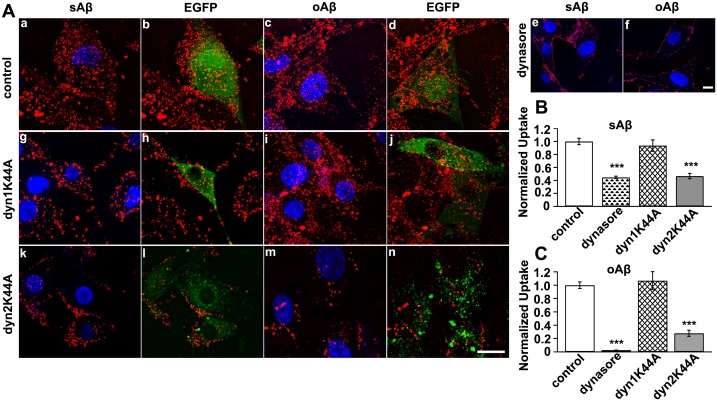
Dynamin 2 participated in Aβ42 uptake by U87 cells. (A) The uptake of Aβ42 species by U87 cells were affected by Dynasore and dominant-negative dynamin2. U87 cells were transfected with an empty vector (a–d), pre-treated with dynasore (e, f) or transfected with GFP-tagged Dyn K44A variants (g–n) followed by incubation with sAβ42 or oAβ42 for 2 h at 37°C. Subsequently, cells were fixed and analyzed uptake of each Aβ42 using confocal microscopy as indicated. Nuclei (blue) were stained with DAPI. Scale bar is 20 µm. (B, C) Quantification of internalized fluorescence-conjugated sAβ42 (B) or oAβ42 (C) in U87 cells based on fluorescence intensity measurements. Values obtained from dynasore-treated or Dyn K44A-expressing cells were normalized to those from mock-treated cells or mock-transfected cells under similar conditions. Results represented the average ± SEM of >25 cells measured in each of three independent experiments. ***p<0.001, t-test.

The results described above reflected the characteristics of sAβ42 and oAβ42 uptake by U87 cells. Furthermore, we explored the dynamin dependence of these processes. The best-studied cellular function of dynamin is its involvement in clathrin-mediated endocytosis. However, dynamin has been implicated in several other membrane-trafficking events, including caveolae-mediated and noncaveolar clathrin-independent endocytic pathways, phagocytosis, macropinocytosis, and trafficking from the *trans*-Golgi network [31]. We first used Dynasore, a fast-acting, cell-permeable, small molecule to inhibit dynamin GTPases (Dyn1 and Dyn2) before incubating cells with Aβ42 peptides [32]. The internalization of sAβ42 and oAβ42 was strongly blocked after Dynasore treatment (Figure 3A, a–f). Only sparse fluro-sAβ42 punctate fluorescence was observed within these cells, and plasma membrane-deposited fluro-sAβ42 indicated that surface-bound sAβ42 could not be transported into these cell. Furthermore, oAβ42 entry was almost entirely blocked, as more fluro-oAβ42 molecules accumulated around the plasma membrane and no punctate fluorescence was observed in these cells (Figure 3A, e–f). Quantifying the fluorescence intensity results confirmed the inhibition of both Aβ42 peptides from being taken up into U87 cells and suggested an essential role for dynamin in their endocytosis (Figure 3B and C).

Mammals express three Dyn isoforms in a tissue-specific manner. Dynamin 1 (Dyn1) is neuron-specific, dynamin 2 (Dyn2) is ubiquitously expressed, and dynamin 3 (Dyn 3) is exclusively expressed in the testis, lung, and brain. This suggests that distinct isoforms may mediate specific cellular functions [33]. To test the dependence of Aβ species endocytosis on Dyn isoforms, we examined the inhibitory effects of Dyn1 and Dyn2 GTPase mutants. Expression of a Dyn 1 dominant-negative K44A mutant had no effect on the uptake of sAβ42 or oAβ42, whereas a Dyn 2 K44A mutant effectively inhibited the internalization of both Aβ42 species by U87 cells (Figure 3A, g–n). Quantitative fluorescence intensity results showed that about 53% of sAβ42 and 72% of oAβ42 uptake was reduced (Figure 3B and C). These findings suggested that Dyn2 function was required for the macropinocytic internalization of Aβ42 species in U87 cells.

### LRP1 is Differentially Involved in the Clearance of Extracellular Monomeric and Oligomeric Aβ42 by U87 Cells

The low-density lipoprotein receptor (LDLR) family of receptors is proteins that have similar structural characteristics and have various important endocytic and signaling functions. Members of this family include LDLR, LRP1, LRP2, very-low density lipoprotein receptor (VLDLR), and apolipoprotein E receptor 2 (ApoER2) [34]. LDLR/LRP1 involvement in Aβ internalization by astrocytes is controversial, in that previous studies did not examine Aβ internalization by astrocytes [4]. Thus, we investigated how altering LRP1 levels might affect the uptake of Aβ into U87 cells.

**Figure 4:**
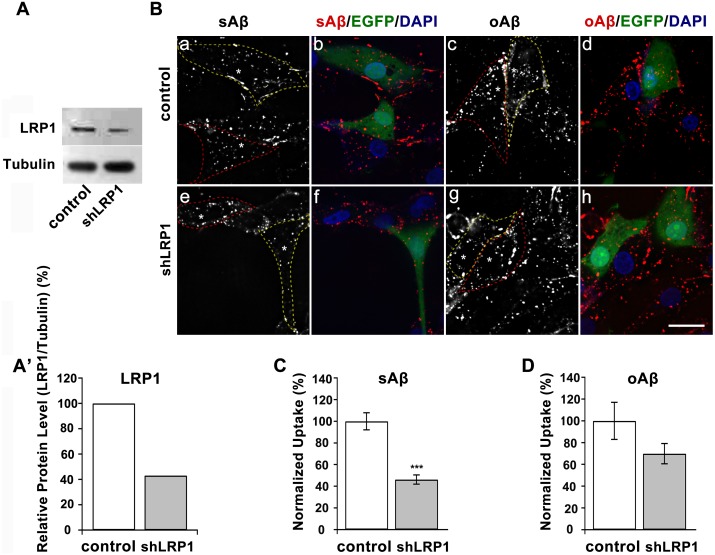
LRP1 receptor dependence of Aβ42 internalization by U87 astrocytic cells. (A, A’) Knockdown of LRP1 levels in U87 cells were measured by immunoblot. Tubulin served as a loading control. (B) Fluorescent micrographs of U87 cells treated with EGFP-tagged vehicle or LRP1 shRNA for 72 h. Cells were incubated with fluorescence-conjugated sAβ42 or oAβ42 for 2 h at 37°C. DAPI was used to stain nuclei. Images were taken by confocal microscopy. Cell contours were outlined for plasmids-expressing positive (yellow dash line) and negative (red dash line) cells, respectively, and asterisks show the nuclei. Scale bar is 20 µm. (C, D) Quantitation of internalized fluorescence-conjugated sAβ42 (C) or oAβ42 (D) in U87 cells after treatment with shLRP1 based on fluorescence intensity measurements. Results represented the average ± SEM, n>23. ***p<0.001, t-test.

U87 cells were transfected with either a vehicle control or LRP1-targeted shRNA and used for analysis 72 h after transfection. The production of LRP1 protein was knocked down to 42%, as confirmed by Western blotting (Figure 4A and A’). When LRP1-suppressed U87 cells were treated with fluro-tagged Aβ42 peptides for 2 h at 37°C, sAβ42 internalization was reduced by about 55% of that in control cells, while a less reduction (30%) in oAβ42 internalization was detected under these conditions (Figure 4B–D). LRP1 involvement in the uptake of sAβ by neural and nonneural cells has been extensively investigated, whereas whether there are any Aβ receptors that mediate these oligomers internalization in neural cells is debatable. Taken together, our results indicate that in U87 cells, although monomeric and oligomeric Aβ42 shared some common properties in their endocytic pathways, oligomers have a priority for entry, which we speculate maybe related to their different dependence on LRP1 receptors.

### Internalized sAβ42 and oAβ42 are Rapidly Transported to Lysosomes through an Endolytic Pathway in U87 Cells

**Figure 5:**
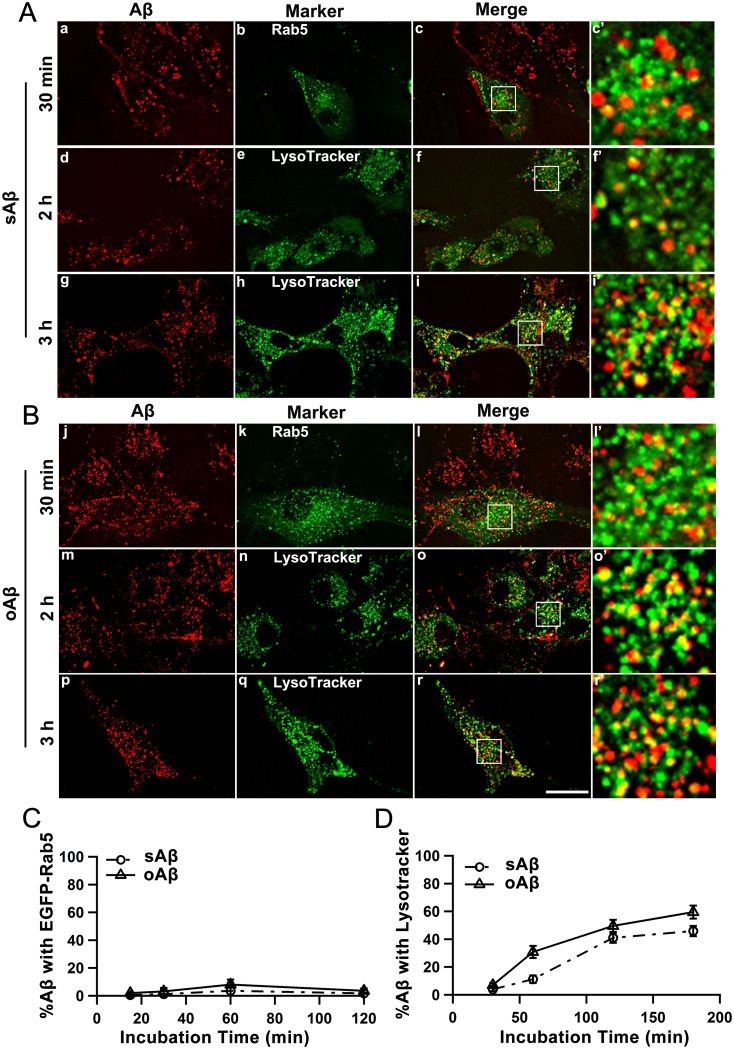
Internalized Aβ42 peptides were rapidly transported to lysosomes through an endolytic pathway. U87 cells were transfected with EGFP-Rab5 to mark early endosomes (a–c and j–l) or stained with LysoTracker Green to mark lysosomes (d–i and m–r). Cells were incubated with 0.4 µM sAβ42 (A) or oAβ42 (B) for various durations and live-cell images were taken by confocal microsopy. Scale bar is 20 µm. (C, D) Quantification of the Aβ42 peptides transport over time. The percentages of colocalization of Aβ42 peptides with EGFP-Rab5 (C) and LysoTracker Green (D) were analyzed and plotted. The error bars represent the average ± SEM of n>15 cells (C) and >25 cells (D).

Previous work with microglial cells proposed that sAβ42 was transferred to late endosomes and lysosomes by a direct fusion of the macropinocytic vesicles to these late endolytic vesicles [11]. We examined the dynamic subcellular destinations of sAβ42 and oAβ42 peptides after their uptake to observe the trafficking routes. U87 cells were treated for the indicated times with fluro-tagged Aβ42 peptides (Figure 5 and Figure S2–S5). Using EGFP-Rab5 as an early endosome marker, we observed that the fractions of both peptides in classical early endosomes were constantly very low (Figure S2 and S3 and Figure 5C), indicating most of the Aβ42 peptides did not transit through the early endosomes. However, the fraction of sAβ42 and oAβ42 localized to vesicles that were positive for Lysotracker Green staining increased with prolonged Aβ42 treating time (Figure S4 and S5 and Figure 5D), which suggested their trafficking to lysosomes. Taken together, these data suggested that after endocytosis, both sAβ42 and oAβ42 species were transferred to the lysosome compartments most probably by direct fusion of vesicles to the late endolytic compartments.

### Fluorescently Labeled sAβ42 and oAβ42 are Subjected to Proteolysis by Lysosomes in U87 Cells

**Figure 6:**
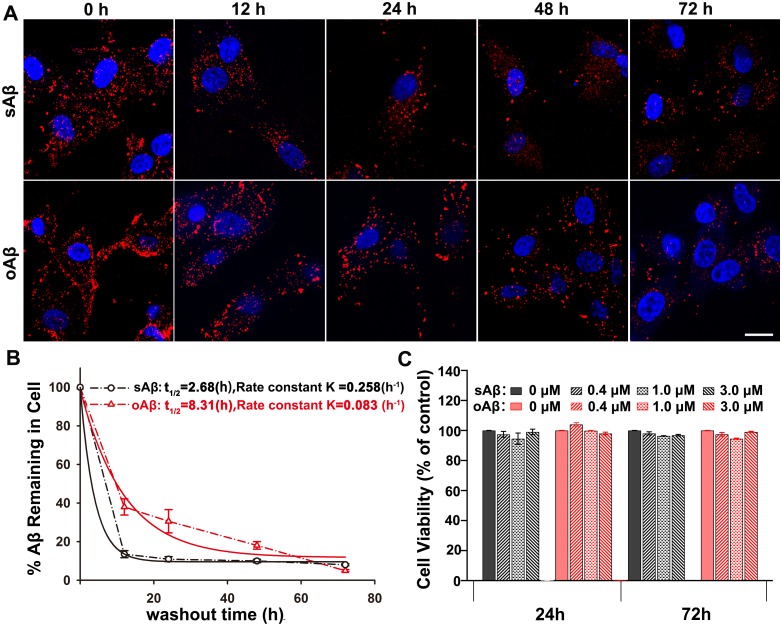
Fluro-tagged Aβ42s were proteolytically degraded by U87 cells without inhibitory effects on cell growth. (A, B) The degradation of labeled sAβ42 or oAβ42 peptides in U87 cells was examined using fluorescence microscopy. Cells were allowed to internalize 0.4 µM Aβ42 species for 2 h, followed by washing out and incubation for additional various durations in medium lacking Aβ42. (A) Representative images of the intracellularly accumulated HiLyte Fluor555-labeled sAβ42 or oAβ42 at different checking time points. Nuclei (blue) were stained with DAPI. Scale bar is 20 µm. (B) The remaining of sAβ42 and oAβ42 at different time points after internalization into U87 cells were plotted. The time points were corresponded to those in (A). The values were obtained by quantifying the remaining average fluorescence intensity of HiLyte Fluor555-labeled sAβ42 or oAβ42 at different time point, relative to that at t = 0 h, and were calculated based on the representative images shown in (A). The data were fitted with the exponential decay curves. Results represented the average ± SEM. At least 8 cells from 3 independent experiments were used to calculate for each time-point**.** (C) U87 cells were treated with sAβ42 or oAβ42 of indicated concentration for 24 h or 72 h, and cell viability was measured by MTS. The mean of three analyses were shown. No significant differences were detected for each condition. P≥0.01, t-test.

Using fluorescently-labeled monomeric and oligomeric Aβ42 peptides, we examined their internalization and intracellular trafficking in U87 cells. This showed that both species were actively taken up and rapidly accumulated within lysosomes. Furthermore, we examined whether the accumulated Aβ species were subjected to proteolysis in the lysosomes of U87 cells. Cells were first incubated with 0.4 µM fluro-tagged sAβ42 or oAβ42 for 2 h, after which the medium was replaced with fresh DMEM. After chasing for different times, cells were fixed and microscopically monitored for their intracellular levels of Aβ species (Figure 6A and B). The representative photomicrographs in Figure 6A illustrate an obvious decrease in intracellularly accumulated punctate fluorescence for both Aβ42 peptides 12 h postwashout of these cells. By quantifying the average integrated fluro-Aβ42 peptides in cells, 14% of sAβ42 and 38% of oAβ42 remained at this time, which indicated the rapid clearance capability of U87 cells for both Aβ species (Figure 6B). In addition, we examined the time courses for degradation of both Aβ species by extending the chasing time after internalization of the Aβ peptides. We observed that at earlier stages (up to 48 h), oAβ42 degraded much slower than sAβ42 by U87 cells, while both species were ultimately efficiently proteolyzed, with sAβ42 remaining of about 8% and oAβ42 remaining of about 5% 72 h after internalization. As shown in Figure 6B, the degradation of both Aβ42 peptides in U87 cells were consistent with a first-order decay process. By fitting data using a single-phase exponential decay equation, the rate-constants are 0.258 h^−1^ for sAβ42 and 0.083 h^−1^ for oAβ42, respectively. The half-times (t_0.5_) for sAβ42 and oAβ42 were calculated to be 2.68 h and 8.31 h, respectively.

It has been suggested that Aβ42, the principal Aβ species in senile plaques, once internalized into neurons, can accumulate within the endosomal–lysosomal system as insoluble aggregates and subsequently trigger neuronal death by compromising lysosomal membrane impermeability [35]. Thus, we examined the effect of Aβ42 on U87 cell viability. Cells were incubated with sAβ42 or oAβ42 and cell viability was determined by MTS assay. Untreated cells were used as a negative control. These results showed that U87 cell growth was not inhibited by either Aβ42 species, even after 72 h (Figure 6C).

Wang *et al*. reported that, as different from the neural cell line SH-SY5Y, astrocytic U87 cells were not induced to undergo apoptosis, even after treatment with 20 µM Aβ, but their viability did decrease because of autophagic cell death [36]. We assumed that U87 cells tolerated the toxic effects of Aβ42 species better than the neurons, particularly because we used relatively low concentrations of Aβ peptides, which could be rapidly cleared by U87 cells and produced negligible cellular toxicity. This property assigns a neuronal protection role to astrocytes because they can promptly clear Aβ peptides from the extracellular milieu, which subsequently maintains the dynamic Aβ balance, also In addition, this suggests an attractive therapeutic target by promoting astroglial Aβ endocytosis and degradation.

## Discussion

Aβ has attracted considerable interest in the field of AD research. Its effects at the cellular level and within the nervous system have been pressing issues for those attempting to develop diagnostic and therapeutic approaches. Vaccines to enhance Aβ clearance are currently under investigation in several clinical trials [37]. However, the mechanisms underlying constitutive and enhanced clearance have not been completely elucidated.

Several pathways for Aβ clearance have been suggested, including (1) Aβ clearance through the blood-brain barrier, (2) extracellular degradation by proteolytic enzymes, and (3) Aβ uptake and degradation by glial cells. Wyss-Coray *et al*. [38] provided direct evidence for a role by astrocytes in Aβ degradation and suggested that there were defects in astroglial clearance of Aβ in AD pathogenesis. This suggested that treatments that increased the removal of Aβ by astrocytes may be a means to reduce the neurodegeneration associated with AD. However, compared with neurons and microglial cells, our understanding of human astrocyte-mediated Aβ internalization and consequences is limited. Our study results should aid in understanding the physiological mechanisms underlying astrocyte clearance of toxic Aβ with different aggregation states.

### Mechanisms of Aβ Internalization in Neurons and Glial Cells

Similar to numerous other cell types, neurons have several major endocytic pathways, including clathrin-dependent, caveolae-dependent, and noncaveolar clathrin-independent pathways. Until recently, clathrin-mediated endocytosis [35], [39] was considered to be the major mechanism of Aβ internalization. However, several other clathrin-independent processes that may mediate Aβ uptake, such as nonsaturable and nonendocytotic uptake, dynamin-dependent and cholesterol-sensitive pathways [40], [41], dynamin-mediated and RhoA-regulated process [42], have been proposed. In addition, microglial cells have been reported to mediate the clearance of fibrillar Aβ through receptor-mediated phagocytosis [9] and can internalize sAβ through a fluid phase macropinocytic mechanism [11].

Previous studies on astroglial cells mainly focused on their mediation of neuronal-glial interactions and the endocytic pathways in astroglial cells are poorly understood [43]. There has been little work done on the internalization pathways for Aβ entry into astrocytes. In this study, we systematically characterized the internalization pathways for the entry of sAβ42 and oAβ42 into U87 cells. We confirmed that a clathrin-independent macropinocytic pathway was responsible for the uptake of both peptides by U87 cells. The capacity of U87 cells to internalize oAβ exceeded that for sAβ, which suggested the importance to clear primary aggregated Aβ (oAβ) by astroglial cells and subsequently protect neurons. We observed that the dynamin 2 and not the dynamin 1 isoform played an important role of clipping away endocytic vesicles from the astroglial cell plasma membrane.

A number of candidate receptors have been suggested to regulate soluble and fibrillar Aβ clearance by microglial cells, including scavenger receptors, Toll-like receptors, and others [44]. However, the cell receptors that facilitate Aβ uptake and clearance by astrocytes have not been extensively characterized. Previous results suggested that Aβ clearance by astrocytes required both apoE and an unknown receptor from the LDLR family. Basak *et al*. [45] highlighted the importance of LDLR in regulating the uptake and clearance of soluble Aβ by astrocytes. LRP1, which is another important member of the LDLR family, has been shown to mediate the metabolism of Aβ in neurons and brain vessels [39], [46]. However, no study has directly determined whether LRP1 is involved in Aβ uptake and degradation in astrocytes.

In our current study, we assessed the effects of down-regulating LRP1 expression by shRNA on astrocytes internalization of sAβ42 and oAβ42. Our results suggest that LRP1 mediates the association of sAβ42 with the cell surface, after which these complexes are internalized into cells through a clathrin-independent macropinocytic pathway. However, the internalization of oAβ42 peptides by U87 cells notably appeared to be unaffected by LRP1 knockdown. A recent report [47] emphasized that only soluble, untreated Aβ42 but not aggregated or oligomeric Aβ, increased apoE protein levels in mouse primary astrocytes, which implied that apoE may mediate a different LRP1 dependence for sAβ42 and oAβ42 internalization in astrocytes. Additional work will be necessary to identify whether there are specific receptors for oAβ42 binding in astrocytes. We speculate that the distinct involvement of receptors may determine the different internalization capacities by astrocytes for sAβ and oAβ.

There is increasing evidence for clathrin- and caveolin-independent pathways for mediating ligand-induced endocytosis. The large GTPase dynamin is involved in both clathin-dependent and -independent pathways. Our results not only enhance our understanding of the mechanisms underlying Aβ peptides internalization by astroglial cells, but also provide support for dynamin involvement in clathrin-independent macropinocytosis, which may be cell type-dependent.

### Consequences of Internalized Aβ, Associated Cell Death, and Dysfunction in Neurons and Glial Cells

sAβ peptides can be dynamically internalized by cells in the CNS. However, the consequences of the internalized Aβ in distinct neurons are different. In neurons, although internalized Aβ is transported within the endosomal system to multivesicular bodies (MVBs) or lysosomes, Aβ is poorly degraded, which may be because of the formation of protease resistant aggregates. Intraneuronal accumulated Aβ has dramatic consequences, such as causing mitochondrial dysfunction or loss of lysosomal membrane impermeability, and leakage of lysosome contents, which eventually cause neuronal apoptosis and necrosis [48], [49]. Abnormal endosomes have been detected in Down syndrome and Niemann-Pick type C in which Aβ peptides intracellularly accumulate [50].

Because microglial cells function as tissue macrophages in the brain and are primary immune effectors within the CNS, they are responsible for fibrillar Aβ clearance. Mandrekar *et al*. [11] reported that microglial cells internalized sAβ peptides; however, they bypassed early endosomes and were rapidly trafficked into late endolysosomal compartments where they were subject to degradation. The capacity for oAβ uptake and degradation by microglial cells was unknown. To the best of our knowledge, our study is the first to examine the intracellular itineraries of monomeric and oligomeric Aβ42 peptides after their uptake by U87 astrocytic cells. We showed that both species were rapidly transferred to lysosomes through an endolytic pathway, most probably bypass the early endosomes. Even at 12 h postinternalization, U87 cells evidently exhibited decreased fluro-sAβ42 and -oAβ42 levels in our imaging experiments, which indicated that Aβ42 degradation was extremely efficient. In particular, this was the case for monomeric peptides. For the oligomers, it is tempting to speculate that the preference of U87 cells for oligomeric Aβ42 results in a relatively high concentration of peptides in lysosomes. This may delay their initial degradation, although this does not attenuate their ultimate thorough degradation.

In addition, we did not observe any obvious reductions in cell viability. This was different from what has been observed with neurons but similar to what is viewed with microglial cells. Thus, we speculate that this is because of the distinct destinies of lysosome accumulated Aβs in different cell types. However, the concentrations of oligomeric Aβ that we used were extremely close to those at physiological conditions, i.e., nanomolar concentrations based on previous reports [51], [52] and not on high concentrations (>10 µM) used in other reports. Our data further suggest that astrocytes play an important role in the clearance of the normally produced aggregates of Aβ so as to maintain Aβ steady-state in the normal brain.

In conclusion, we believe that dissecting the molecular pathways responsible for Aβ internalization by astrocytes, and the mechanisms involved in their proteolytic degradation will suggest new therapeutic strategies for the effective clearance of brain Aβ.

## Supporting Information

Figure S1 U87 cells were incubated with HiLyte Fluor555-labeled sAβ42 to screen optimal experimental conditions. (A) U87 cells were incubated with 5 µM sAβ42 for various durations at 37°C. Cells were then fixed and uptake of sAβ42 was analyzed by confocal microscopy. Nuclei (blue) were stained with DAPI. Results showed that at earlier stages, such as at 5 min, most of the molecules just bound and accumulated around the plasma membrane, while along longer duration of incubation, sAβ42s were eventually internalized into cells. (B) The effect of Aβ42 concentration on internalization was further examined. Results showed that even at 0.4 µM, a submicromolar concentration, the intracellularly accumulated sAβ42s were obvious, while at higher concentrations, these molecules probably tend to aggregate. We finally set the concentration to be 0.4 µM and the incubation time to be 2 h for all subsequent internalization experiments. Scale bar is 20 µm.
(TIF)

Figure S2 Internalized sAβ42 was not transported through early endosome. U87 cells were transfected with EGFP-Rab5, a marker of early endosomes, and incubated with 0.4 µM sAβ42 for 15 min, 30 min, 1 h, or 2 h, respectively. Live-cell images were taken by confocal microsopy. The images showed that a low fraction of sAβ42 was transported into early endosome after internalization. Scale bar is 20 µm.
(TIF)

Figure S3 Internalized oAβ42 was not transported through early endosome. U87 cells were transfected with EGFP-Rab5, which marked early endosomes, and incubated with 0.4 µM oAβ42 for 15 min, 30 min, 1 h or 2 h, respectively. Live-cell images were taken by confocal microsopy. The images indicated that a low fraction of oAβ42 passed through the early endosome after internalization. Scale bar is 20 µm.
(TIF)

Figure S4 After internalization, sAβ42 was rapidly transported to lysosomes. U87 cells were incubated with 0.4 µM sAβ42 for 30 min, 1 h, 2 h, or 3 h, respectively, and stained with LysoTracker Green to mark lysosomes. Live-cell images were taken by confocal microsopy. As shown, very little amount of sAβ42 were localized to lysosomes at the time point of 30 min, while these molecules accumulated into lysosomes eventually. Scale bar is 20 µm.
(TIF)

Figure S5 oAβ42 was rapidly transported to lysosomes after internalization. U87 cells were incubated with 0.4 µM oAβ42 for 30 min, 1 h, 2 h, or 3 h, respectively, and stained with LysoTracker Green to mark lysosomes. Live-cell images were taken by confocal microsopy. As shown, very little amount of oAβ42 were localized to lysosomes at the time point of 30 min, while these molecules accumulated into lysosomes eventually. Scale bar is 20 µm.
(TIF)
