# Supplementary figures and images for: Mechanisms of U87 Astrocytoma Cell Uptake and Trafficking of Monomeric versus Protofibril Alzheimer’s Disease Amyloid-β Proteins
Source: PLoS One. 2014 Jun 18;9(6):e99939. doi: 10.1371/journal.pone.0099939 (PMC4062444; doi:10.1371/journal.pone.0099939)

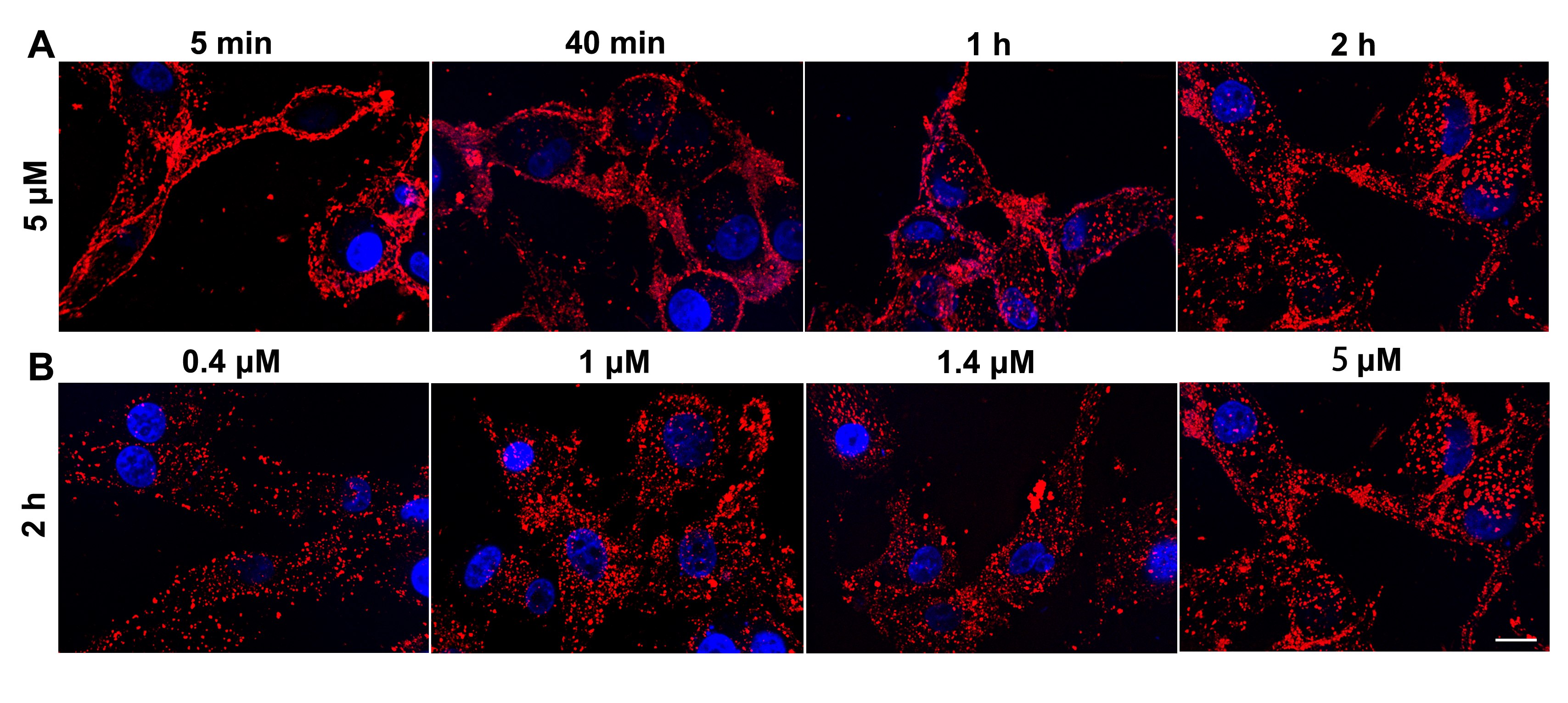

Supplement: Figure S1 — U87 cells were incubated with HiLyte Fluor555-labeled sAβ42 to screen optimal experimental conditions. (A) U87 cells were incubated with 5 µM sAβ42 for various durations at 37°C. Cells were then fixed and uptake of sAβ42 was analyzed by confocal microscopy. Nuclei (blue) were stained with DAPI. Results showed that at earlier stages, such as at 5 min, most of the molecules just bound and accumulated around the plasma membrane, while along longer duration of incubation, sAβ42s were eventually internalized into cells. (B) The effect of Aβ42 concentration on internalization was further examined. Results showed that even at 0.4 µM, a submicromolar concentration, the intracellularly accumulated sAβ42s were obvious, while at higher concentrations, these molecules probably tend to aggregate. We finally set the concentration to be 0.4 µM and the incubation time to be 2 h for all subsequent internalization experiments. Scale bar is 20 µm. (TIF) [file pone.0099939.s001.tif]

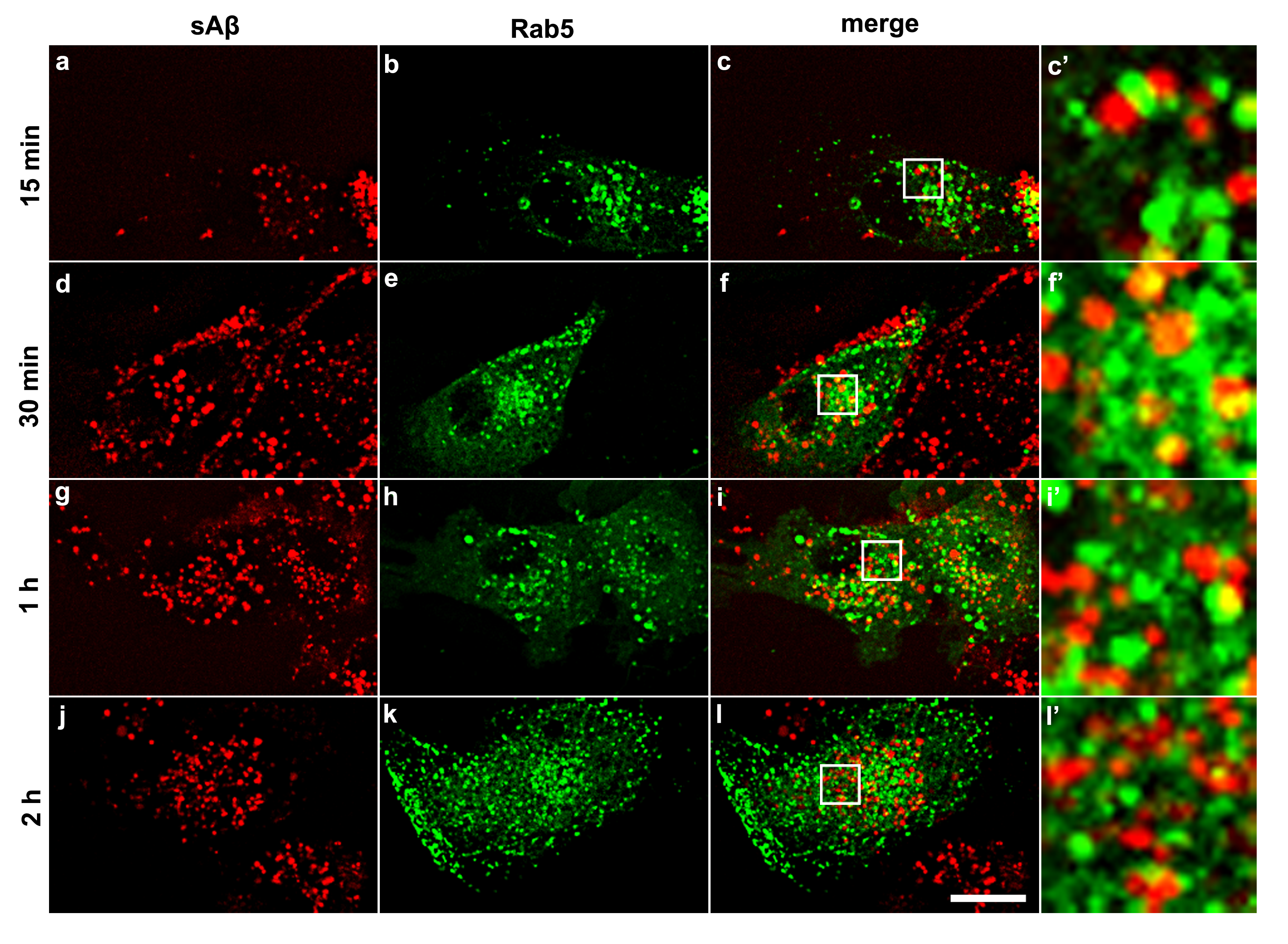

Supplement: Figure S2 — Internalized sAβ42 was not transported through early endosome. U87 cells were transfected with EGFP-Rab5, a marker of early endosomes, and incubated with 0.4 µM sAβ42 for 15 min, 30 min, 1 h, or 2 h, respectively. Live-cell images were taken by confocal microsopy. The images showed that a low fraction of sAβ42 was transported into early endosome after internalization. Scale bar is 20 µm. (TIF) [file pone.0099939.s002.tif]

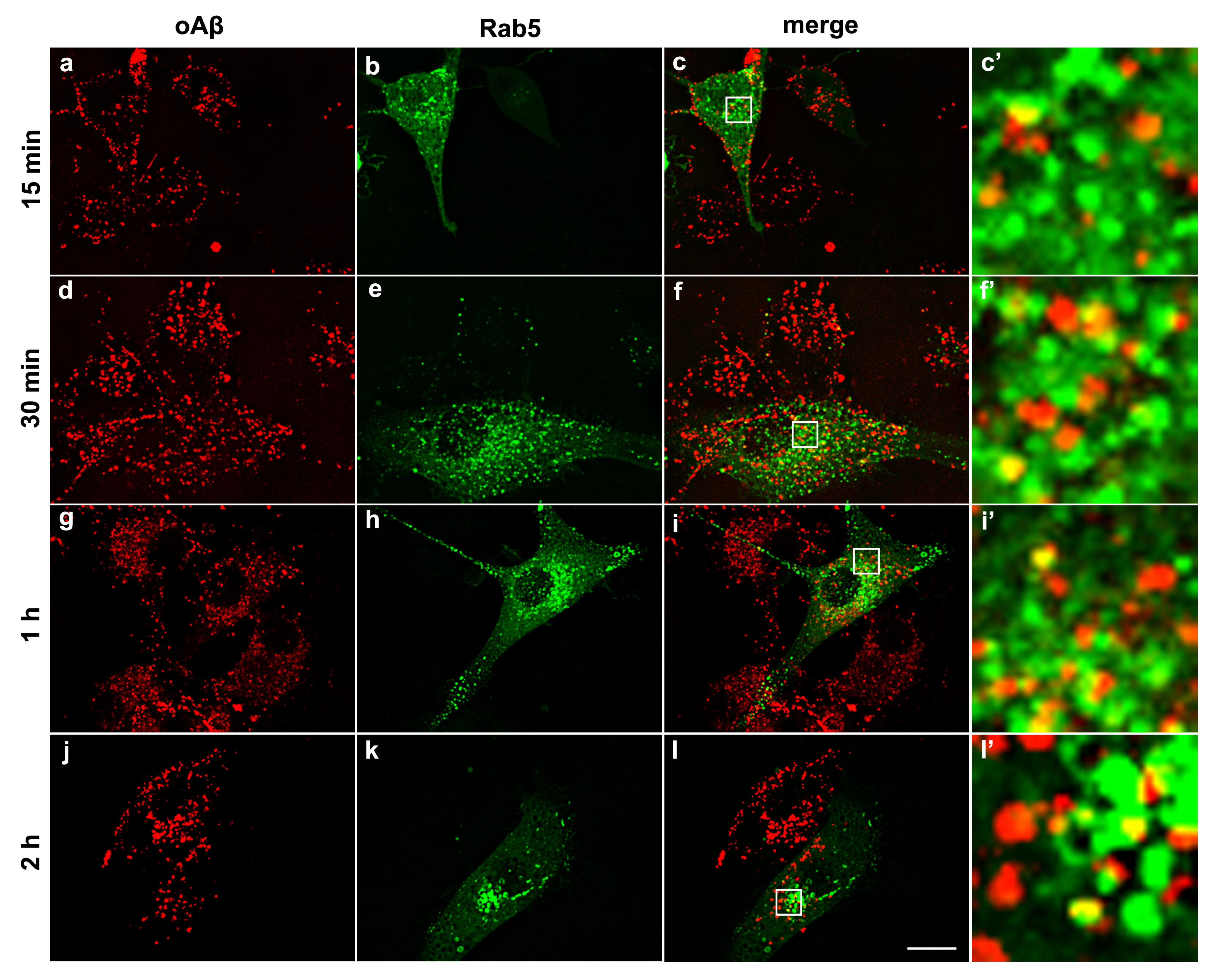

Supplement: Figure S3 — Internalized oAβ42 was not transported through early endosome. U87 cells were transfected with EGFP-Rab5, which marked early endosomes, and incubated with 0.4 µM oAβ42 for 15 min, 30 min, 1 h or 2 h, respectively. Live-cell images were taken by confocal microsopy. The images indicated that a low fraction of oAβ42 passed through the early endosome after internalization. Scale bar is 20 µm. (TIF) [file pone.0099939.s003.tif]

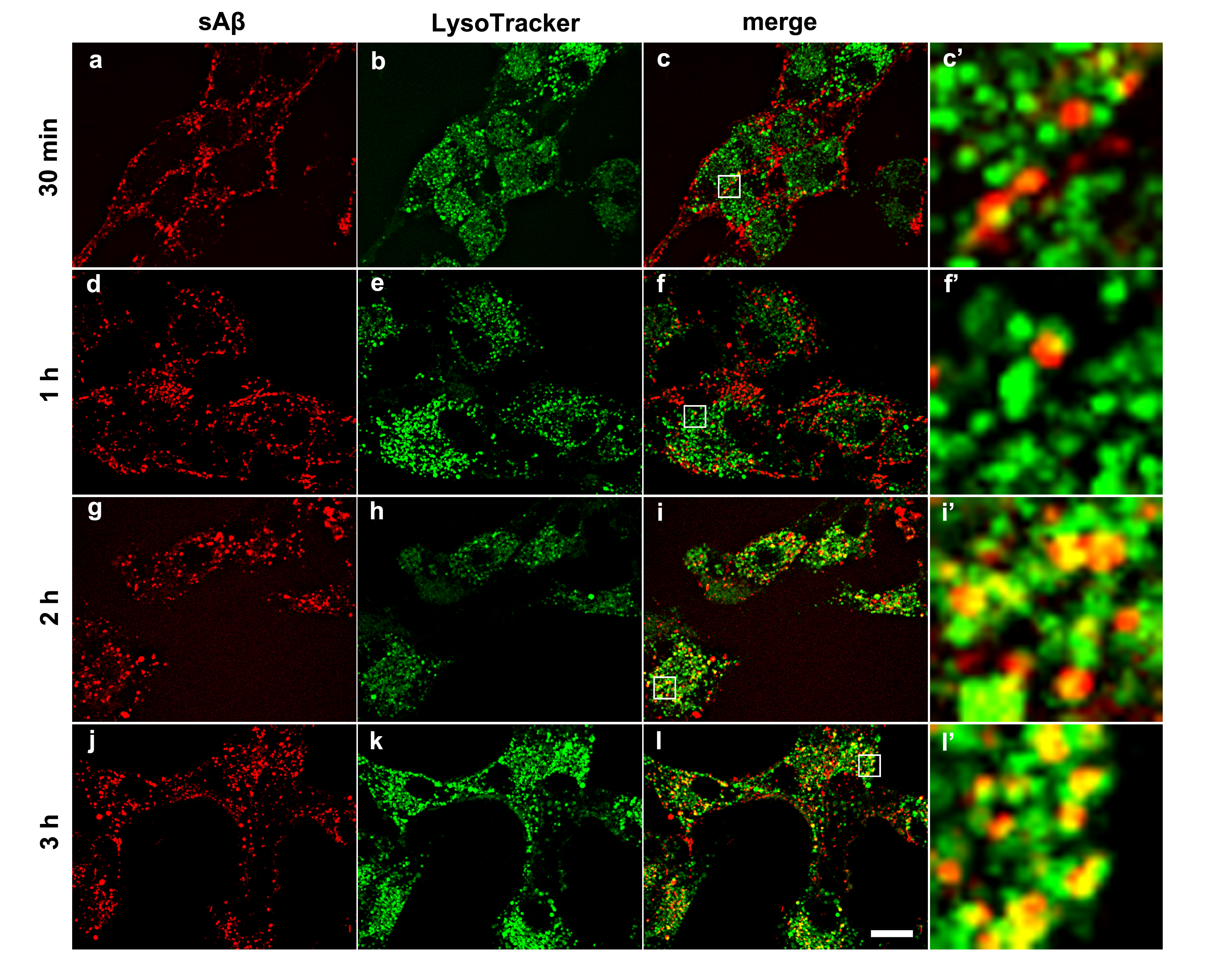

Supplement: Figure S4 — After internalization, sAβ42 was rapidly transported to lysosomes. U87 cells were incubated with 0.4 µM sAβ42 for 30 min, 1 h, 2 h, or 3 h, respectively, and stained with LysoTracker Green to mark lysosomes. Live-cell images were taken by confocal microsopy. As shown, very little amount of sAβ42 were localized to lysosomes at the time point of 30 min, while these molecules accumulated into lysosomes eventually. Scale bar is 20 µm. (TIF) [file pone.0099939.s004.tif]

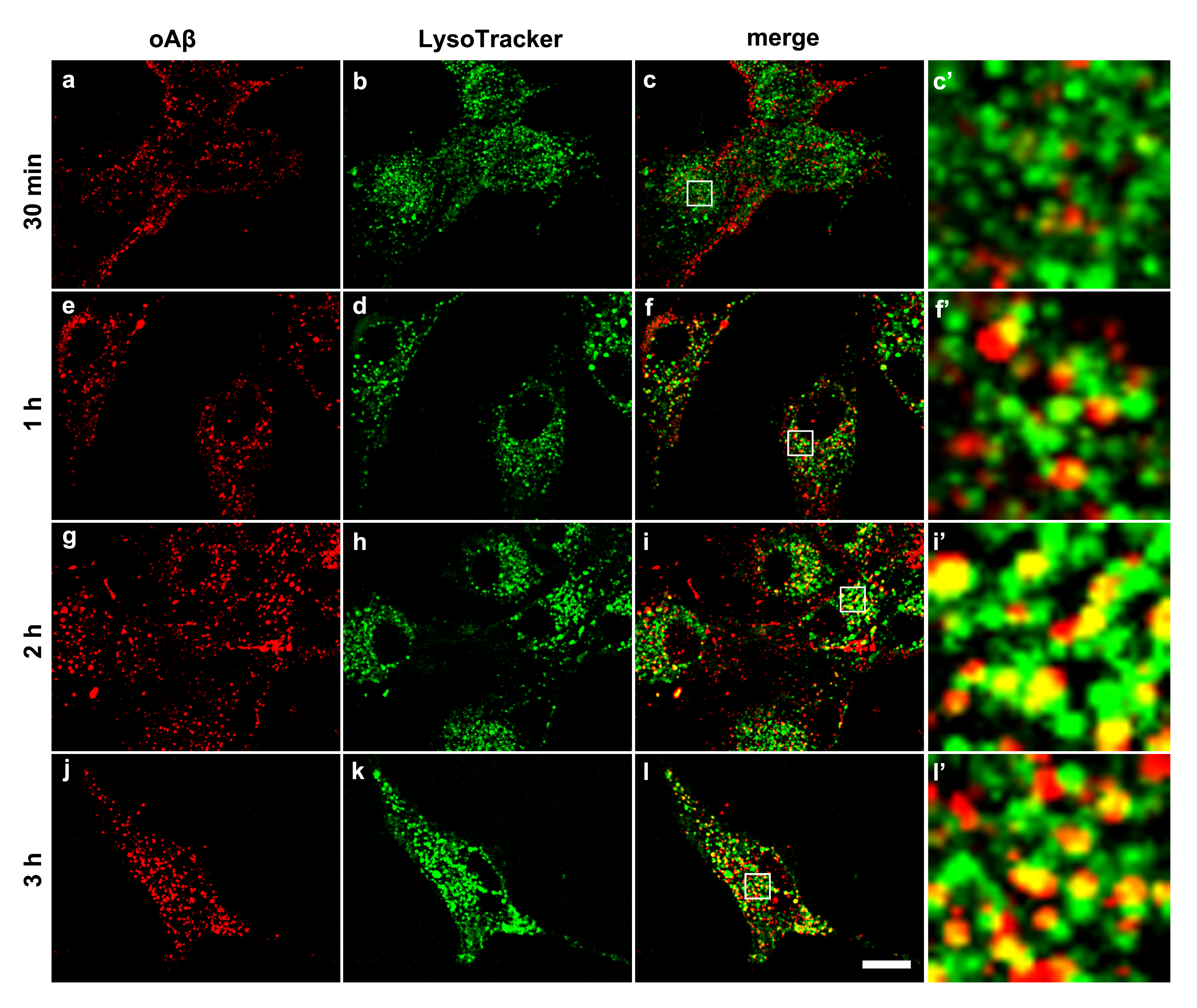

Supplement: Figure S5 — oAβ42 was rapidly transported to lysosomes after internalization. U87 cells were incubated with 0.4 µM oAβ42 for 30 min, 1 h, 2 h, or 3 h, respectively, and stained with LysoTracker Green to mark lysosomes. Live-cell images were taken by confocal microsopy. As shown, very little amount of oAβ42 were localized to lysosomes at the time point of 30 min, while these molecules accumulated into lysosomes eventually. Scale bar is 20 µm. (TIF) [file pone.0099939.s005.tif]
